# Aortic Stenosis-Associated Cardiac Damage: A Comparison Between Patients Treated with Surgery and Transcatheter Aortic Valve Replacement

**DOI:** 10.3390/jcm15051961

**Published:** 2026-03-04

**Authors:** Ricardo Román, Fabián Islas, Patrick O’Neill-González, Carlos E. Gil, Manuel Carnero, Pilar Jiménez-Quevedo, Luis Nombela-Franco, Daniel Pérez-Camargo, Lourdes Montero-Cruces, María Rivadeneira-Ruiz, Sandra Gil-Abizanda, Eduardo Pozo, Carmen Olmos

**Affiliations:** 1 Instituto Cardiovascular, Hospital Clínico San Carlos, Instituto de Investigación Sanitaria del Hospital Clínico San Carlos (IdISSC), 28040 Madrid, Spain; 2 Hospital Universitario de La Zarzuela, 28023 Madrid, Spain; 3 Facultad de Medicina, Salud y Deportes, Universidad Europea de Madrid, 28670 Madrid, Spain

**Keywords:** transcatheter aortic valve replacement, surgical aortic valve replacement, aortic stenosis, cardiac damage, staging, and echocardiography

## Abstract

**Background/Objectives**: In patients with severe aortic stenosis (AS), extra valvular cardiac damage is associated with outcomes following intervention. We aimed to analyze differences in AS-related cardiac damage between surgical aortic valve replacement (SAVR) and transcatheter aortic valve replacement (TAVR) patients in a contemporary cohort of patients with severe AS. **Methods**: Patients who underwent SAVR or TAVR in a tertiary care center from 2017 to 2022 were included in this retrospective analysis. Clinical and echocardiographic parameters before surgery were compared, and patients were classified according to two available cardiac damage staging systems. Additionally, all-cause mortality 1-year post-intervention was assessed according to these stages. **Results:** Eight hundred and seventy-four patients were included (524 underwent TAVR and 350 underwent SAVR). TAVR patients were significantly older (81.9 ± 6.1 vs. 69.5 ± 9.5 years; *p* < 0.001), more commonly female (52.4% vs. 37.7%; *p* < 0.001), and had higher surgical risk (EuroSCORE II 6.2 ± 7.0 vs. 2.7 ±3.3; *p* < 0.001). Compared with SAVR, patients treated with TAVR had significantly more advanced (right-sided) cardiac damage, both with Généreux (37.8% vs. 23.1%; *p* < 0.001) and Gutiérrez-Ortiz (16.5% vs. 9.0%; *p* = 0.007) staging systems. Moreover, regardless of the type of intervention or the staging system used, mortality was significantly higher in patients with right-sided damage. **Conclusions**: In a contemporary cohort of severe symptomatic AS patients, those treated with TAVR had significantly more extensive cardiac damage compared with those who underwent SAVR. This finding raises the question of when to intervene in patients chosen for TAVR. Earlier intervention, before advanced cardiac damage ensues, might help to improve outcomes.

## 1. Introduction

Since its introduction into clinical practice guidelines as an alternative to surgical intervention in patients with severe aortic stenosis (AS), particularly those at high or intermediate surgical risk, the use of transcatheter aortic valve replacement (TAVR) has increased over the last decade. This change has been paralleled by a decrease in the number of surgical aortic valve replacement (SAVR) procedures performed [1,2].

Current class I recommendations for aortic valve replacement (AVR) are based on the presence of symptoms or left ventricular (LV) systolic dysfunction with a left ventricular ejection fraction (LVEF) < 50% [3], given its poor prognosis [4,5]. However, in recent years, growing evidence of other cardiac remodeling findings as prognostic factors in patients treated with AVR [6], as well as changes (reverse or further remodeling) with intervention [7], has led to the hypothesis of better clinical outcomes with improved timing of intervention based on this extravalvular (extra-aortic valve) cardiac damage.

The first evidence in the literature on the prognostic relevance of extravalvular cardiac damage for short-term all-cause mortality (i.e., 1 year) was reported by Généreux et al. in 2017 [6], who stratified this pre-intervention associated cardiac damage in severe symptomatic AS patients into five stages (0–4) using predominantly echocardiographic parameters. This staging system showed incremental prognostic value over other covariates. Later, this impact was expanded to quality of life [8] and longer follow-up [7,9], as well as other AS groups such as asymptomatic severe [10] and moderate AS [11]. Interestingly, cardiac damage progression or regression post-intervention has also been associated with improved or worsened clinical outcomes, respectively [7,9].

Other more recent cardiac damage staging systems, such as the one proposed by Gutiérrez-Ortiz et al. [12] or modifications of that by Généreux et al. [13], are consistent in that the greater the associated cardiac damage, the greater the mortality during follow-up.

We aimed to assess the differences in AS-related cardiac damage between patients undergoing SAVR and those treated with TAVR in a contemporary cohort of patients with severe symptomatic AS. Additionally, we examined differences in mortality from any cause 1 year after the intervention, by extent of cardiac damage, in both groups.

## 2. Methods

### 2.1. Study Design

Retrospective analysis of a prospective cohort of consecutive patients who underwent either TAVR or SAVR because of severe symptomatic AS, according to current guidelines [3], in a tertiary care center in Spain from January 2017 to May 2022 was conducted. The type of intervention was determined by the treating physician and the Heart Team, taking into account anatomical features, clinical characteristics, frailty, and patients’ preferences.

Relevant clinical and epidemiological information was retrieved from a comprehensive evaluation systematically carried out prior to intervention and included in an ongoing registry. Standardized data collection and unified definitions are used.

Imaging information was obtained from transthoracic echocardiograms performed within 2 months of the intervention with commercially available ultrasound systems, and analyzed by cardiovascular imaging specialists in accordance with established imaging guidelines [14,15,16,17].

The protocol was approved by the local ethics committee and complied with the ethical guidelines of the 1975 Declaration of Helsinki. (CEIC 20.238-E/AS registry).

### 2.2. Cardiac Damage Staging Classification

**Figure 1:**
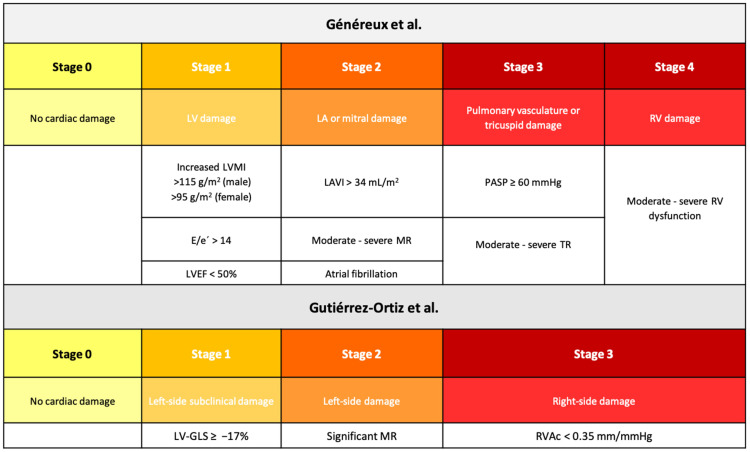
Cardiac damage staging systems in aortic stenosis proposed by Généreux [6] and Gutiérrez-Ortiz [12]. LVEF: left ventricular ejection fraction, LV-GLS: left ventricle global longitudinal strain, LVMI: left ventricle mass index, MR: mitral regurgitation, PASP: pulmonary artery systolic pressure, RVAc: right ventricular–arterial coupling, TR: tricuspid regurgitation.

Using clinical and transthoracic echocardiography data, patients from both TAVR and SAVR groups were classified according to the cardiac damage staging systems proposed by Généreux et al. [6] and Gutiérrez-Ortiz et al. [12] (Figure 1).

### 2.3. Follow-Up and Outcomes

Follow-up began from the date of aortic valve intervention. The primary outcome for the two staging systems was all-cause one-year mortality. Survival data were complete for all patients and obtained from institutional medical records and a regional centralized database covering all public hospitals. In addition, post-discharge mortality was confirmed by the national death register data. Follow-up time was calculated as the difference between the intervention date and the date of death or the last medical contact.

### 2.4. Statistical Analysis

Categorical variables are presented as frequencies and percentages and compared using the χ^2^ test or Fisher’s exact test as appropriate. Continuous data are displayed as mean and standard deviation and compared using the Student’s *t*-test and the nonparametric Mann–Whitney U test when necessary. Data normality was assessed using the Shapiro–Wilk test.

Two sensitivity analyses were performed to evaluate the potential influence of the estimated surgical risk by the EuroSCORE II on the distribution of cardiac damage: the first after excluding patients with high surgical risk, and the second including only patients with low surgical risk.

Cox regression analysis was conducted to identify predictors of all-cause mortality within 1 year of the intervention. Variables significant in univariable analysis (*p* < 0.05) or considered clinically relevant were entered into the multivariable model, with final selection based on minimizing Akaike and Bayesian information criteria. Right-sided damage, as defined by the two different staging systems, was alternatively included in multivariable models. Adjusted hazard ratios (HR) with 95% confidence intervals (CIs) were calculated for each variable included in the model. No significant multicollinearity, assessed using variance inflation factors (VIF < 10), was detected. Proportional hazards assumptions were verified using Schoenfeld residuals. Survival curves were generated with the Kaplan–Meier method, and differences between groups were assessed using the log-rank test.

All tests were two-sided, and differences were considered statistically significant at *p*-values < 0.05. The statistical analysis was done with Stata 17 (StataCorp, College Station, TX, USA).

## 3. Results

### 3.1. Clinical and Epidemiological Characteristics

A total of 874 patients were included: 524 underwent TAVR, and 350 underwent SAVR. A comparison of the clinical characteristics between TAVR and SAVR patients is presented in Table 1. Patients who underwent TAVR were older (81.9 [6.1] vs. 69.5 [9.5] years; *p* < 0.001) and more commonly female (52.4% vs. 37.7%; *p* < 0.001). The prevalence of arterial hypertension, smoking, chronic kidney disease, atrial fibrillation, and obesity was significantly higher in TAVR patients, compared with the SAVR group. In addition, EuroSCORE II was significantly higher in TAVR patients (6.2 [7.0] vs. 2.7 [3.3]; *p* < 0.001). Sensitivity analyses, excluding patients with high surgical risk (EuroSCORE II > 8) and considering only patients with low risk (EuroSCORE II < 4), demonstrated consistent results (Table 2 and Supplementary Table S1, respectively).

### 3.2. Echocardiographic Findings

Patients in the TAVR group had significantly lower left ventricular ejection fraction, higher left ventricular filling pressure (assessed by the E/e’ ratio), and higher left atrial volume index (LAVI) than SAVR patients (Table 3). Left ventricular global longitudinal strain was similar in both groups. Regarding concomitant heart valve disease, significant mitral regurgitation (17.7% vs. 9.8%; *p* = 0.001) and tricuspid regurgitation (15.2% vs. 7.8%; *p* = 0.001) were identified more frequently in patients treated with TAVR. Finally, we documented higher pulmonary artery systolic pressure and worse right ventricular function, measured by tricuspid annular plane systolic excursion (TAPSE) and right ventricular–arterial coupling (RVAc), in the TAVR group.

There was a strong inverse correlation between LAVI and RVAc, both in patients treated with TAVR (*r* = −0.265, *p* < 0.001) and in SAVR patients (*r* = −0.314, *p* < 0.001).

Sensitivity analyses, excluding high surgical risk patients (EuroSCORE II > 8) and considering only low-risk patients (EuroSCORE II < 4), confirmed these findings (Table 4 and Supplementary Table S2, respectively).

### 3.3. Cardiac Damage Staging

**Figure 2:**
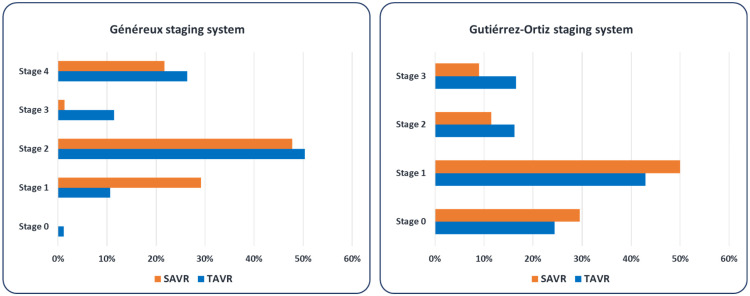
Distribution of patients in the SAVR and TAVR groups based on the cardiac damage staging systems proposed by Généreux [6] and Gutiérrez-Ortiz [12].

Patients were classified according to the two previously mentioned staging systems for extra-aortic valve cardiac damage (Figure 2). Applying the staging system proposed by Généreux et al. [6] to patients treated with SAVR, 29.1% of patients met criteria for Stage 1 (LV damage), 47.8% were in Stage 2 (left atrial or mitral valve damage), 1.4% in Stage 3 (pulmonary artery vasculature or tricuspid valve damage), and 21.7% of patients were in Stage 4 (right ventricular damage). There were no patients in Stage 0. In the same group, Gutiérrez-Ortiz staging system [12] resulted in Stage 0 (no cardiac damage) in 29.5% of patients, Stage 1 (left-side subclinical damage) in 50%, Stage 2 (left-side damage) in 11.5%, and Stage 3 (right-side damage) in 9.0% of patients treated with SAVR.

In the TAVR group, applying the aforementioned classifications yielded the following results: Stage 0 (Généreux): 1.2%; Stage 1: 10.6%; Stage 2: 50.3%; Stage 3: 11.5%; and Stage 4: 26.4%. When using the staging system by Gutiérrez-Ortiz, Stage 0 comprised 24.4% of patients, Stage 1, 42.9%, Stage 2, 16.2%, and Stage 3, 16.5%.

**Figure 3:**
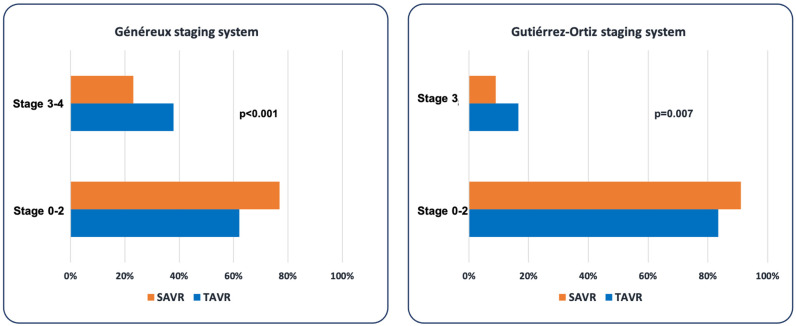
Advanced (right-sided) cardiac damage in the SAVR and TAVR groups based on the cardiac damage staging systems proposed by Généreux and Gutiérrez-Ortiz.

As can be observed, patients treated with TAVR had significantly more advanced cardiac damage (right-side damage) compared with those in the SAVR group, both considering Stages 3 and 4 of Généreux classification (37.8% vs. 23.1%; *p* < 0.001), and Stage 3 of Gutiérrez-Ortiz classification (16.5% vs. 9.0%, *p* = 0.007) (Figure 3).

### 3.4. All-Cause Mortality During Follow-Up

Median follow-up after the intervention was 2.73 (1.81–3.79) years. All-cause mortality at 1 year after the intervention occurred in 17 (4.9%) patients in the SAVR group and in 92 (17.6%) patients in the TAVR group (*p* < 0.001).

**Figure 4:**
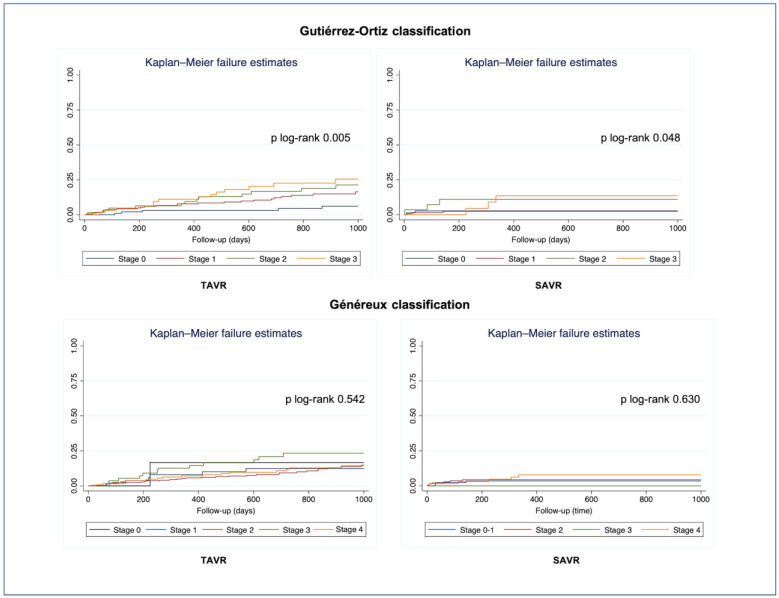
Kaplan–Meier survival curves for all-cause mortality by stage of cardiac damage.

We found a statistically significant stepwise increase in all-cause mortality with greater degrees of cardiac damage, as assessed by the Gutiérrez-Ortiz staging system [12], both in patients treated with SAVR (*p* = 0.005) and in those who underwent TAVR (*p* = 0.048). No significant differences in mortality between stages of cardiac damage were found when applying the Généreux classification in both treatment groups (SAVR, *p* = 0.630; TAVR, *p* = 0.542) (Figure 4).

**Figure 5:**
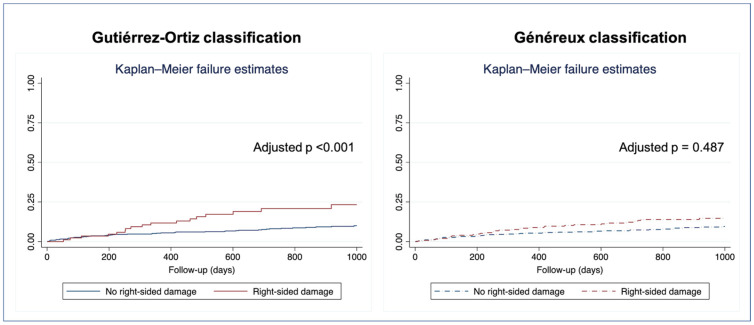
Kaplan–Meier survival curves for all-cause mortality by right-sided cardiac damage.

In multivariable Cox regression analysis (Table 5, Figure 5), right-sided damage, defined as Stage 3 of Gutiérrez et al., was independently associated with one-year all-cause mortality in the whole cohort. Conversely, right-sided cardiac damage, as defined by the Généreux classification, was not independently associated with outcomes.

The score proposed by Gutiérrez et al. showed better discrimination for mortality than the one proposed by Généreux et al. (Harrell’s C index 0.690 vs. 0.585), with an integrated discrimination improvement of 0.035 (*p* < 0.001).

## 4. Discussion

This study presents the results of a contemporary real-life cohort of patients with severe symptomatic AS treated in a tertiary care center. The main findings of our study include: (1) a higher number of TAVR interventions performed, with respect to SAVR, within the predefined time window; (2) TAVR patients were older, more frequently female, had more comorbidities and higher surgical risk; (3) patients treated with TAVR had a more advanced stage of extravalvular cardiac damage, even when analyzing only low-risk patients; and (4) right-sided cardiac damage, assessed by RVAc, was independently associated with one-year mortality in both treatment groups.

TAVR has dramatically changed AS treatment, leading to an increase in percutaneous procedures in recent years and almost doubling their frequency in the intermediate- and low-risk groups [1,2]. In our study, this was reflected in the higher number of TAVR cases, most of which were intermediate risk according to EuroSCORE II.

In this real-world cohort, the type of intervention was determined based on clinical and anatomical features, as well as patients’ preferences, and thus, there is an inherent selection bias. However, in recent years, in the absence of contraindications for one type of intervention over another, the main criterion for choosing the type of aortic valve replacement is age, although the predicted surgical risk is also considered.

The clinical profile of patients included in our study resembles that of patients in pivotal AS intervention trials. As such, patients in our TAVR group were comparable to the population recruited in the intermediate-surgical-risk trial, PARTNER 2A, with predicted surgical risk, mean age, and female percentage quite similar [18]. However, after excluding high-risk patients, the clinical profile of our TAVR group was similar to that reported in recent real-life registries of low-risk populations and pragmatic randomized trials [19,20]. On the other hand, patients in the SAVR group were similar to those in the low-surgical-risk trial PARTNER 3 [21], with similar predicted surgical risk, mean age, and female proportion.

Extravalvular cardiac damage was diagnosed and staged according to two previously published schemes [6,12]. It is interesting to note that from 2017 onwards, an increasing number of studies on this issue have been published. The first work that paved the way for this field was written by Généreux et al. [6]. More recently, these authors have pooled data from the PARTNER 2A, PARTNER 2B, and PARTNER 3 trials, and classified 1974 patients (1180 patients treated with TAVR, and 794 with SAVR) into 5 stages of cardiac damage, encompassing the largest cohort of patients to date in whom cardiac damage prior to intervention is assessed [8]. A comparison of their findings and ours allows us to draw several inferences:

First, in both cohorts, Stage 2 comprised the highest proportion of patients in the TAVR and SAVR groups.

Second, Généreux et al. reported a trend toward a higher proportion of patients with advanced cardiac damage (Stages 3–4) in the TAVR group compared with those treated with SAVR (30% vs. 24%, respectively), a finding we have confirmed in our cohort. Yet the percentage of patients in our TAVR group with right-sided cardiac damage according to this classification was slightly higher (37%). This finding may reflect the interaction and influence of other comorbidities, such as atrial fibrillation or obesity, which were very prevalent in our TAVR cohort and are known to be associated with heart failure with preserved ejection fraction and elevated pulmonary pressures [22,23]. The inverse correlation between LAVI and RVAc found in our cohort further supports this hypothesis and could provide a mechanistic link between elevated left-sided filling pressures and RV–pulmonary arterial uncoupling.

Third, the Gutiérrez-Ortiz staging system [12] also yielded a higher proportion of patients with advanced cardiac damage (Stage 3) in the TAVR group.

The importance of extensive extravalvular cardiac damage has been consistently demonstrated in the literature, with poorer clinical outcomes after AVR at a higher pre-intervention cardiac damage stage [24]. Notably, conflicting evidence may appear with respect to the early Stage 1 (LV damage), as some studies, both in symptomatic severe AS [9] and asymptomatic moderate and severe AS [10], found higher all-cause mortality (compared with Stage 0 or no cardiac damage) only when starting from Stage 2 (cardiac damage beyond the left ventricle). However, these studies only included a small proportion of patients with LV systolic dysfunction (<30%) [9] or did not include them at all [10], so the relevance of LV systolic dysfunction may not have been reliably evaluated.

Beyond this controversy, it is blatantly evident that baseline right-sided damage (pulmonary vasculature, tricuspid valve, or right ventricular damage) has substantial prognostic implications [25,26], independent of cardiac damage progression (sequential or non-sequential) or its association with non-cardiac causes [7]. Furthermore, patients with advanced damage have higher mortality even if they have some degree of cardiac damage improvement post-AVR [7,9].

In our study, we further demonstrated that right-sided cardiac damage, particularly as defined by RVAc, was independently associated with worse outcomes, regardless of the type of intervention (surgical or percutaneous). It is important to note, however, that the highest mortality occurred in TAVR patients with right-sided damage.

In light of these results, it is necessary to address why a specific group of patients is still referred late for treatment of AS, despite the availability of two excellent options for high-, moderate-, and low-risk patients. Eight years after the publication of the first article on cardiac damage staging [6], these tools are still not incorporated into routine clinical practice. Meanwhile, it is becoming increasingly apparent that avoiding advanced cardiac remodeling is crucial to improving clinical outcomes. Hence, we propose implementing cardiac damage staging systems in the clinical setting, as they could be a helpful tool for selecting patients for earlier intervention before advanced damage occurs.

### Limitations

Our work has several limitations that should be acknowledged. As previously mentioned, this is a single-center, retrospective observational study with the inherent limitations of this design. Thus, the mode of intervention was decided by the treating clinicians and the Heart Team, taking into account anatomical and clinical considerations. Nevertheless, it represents the management of a real-life cohort of patients with severe AS in a tertiary care center.

In addition, information on frailty assessment was not available. Furthermore, echocardiographic measurements were performed for clinical use; no reproducibility assessment was done. Although echocardiographic variables were included in the database retrospectively, all measurements were performed prospectively by different operators, experts in cardiovascular imaging, and recorded electronically, reflecting prospective evaluation and quantitation in routine practice with transthoracic echocardiography.

Finally, we are aware that extravalvular cardiac damage is not necessarily related exclusively to AS and may result from other comorbidities, and baseline differences between SAVR and TAVR may lead to residual confounding. However, regardless of the leading cause of cardiac damage, we have found that TAVR patients are treated at more advanced stages and that a higher degree of cardiac damage is associated with worse outcomes. Prospective studies are necessary to evaluate the usefulness of cardiac damage staging as a decision-guiding strategy.

## 5. Conclusions

Real-world data from a contemporary cohort of symptomatic severe AS patients show a higher proportion of extensive cardiac damage in TAVR patients, independently of the surgical risk and the cardiac damage staging system used. This finding, although the proportion of specific comorbidities may influence it, indicates that there is a group of patients in whom AS evolves to a state of maximal cardiac damage without treatment, and might reflect delayed intervention in this large, older, and more vulnerable group.

Thus, we propose that extravalvular cardiac damage stratification, beyond LVEF, although not recommended by current clinical practice guidelines, should be incorporated into the routine management of patients with AS to optimize the timing of intervention, improve clinical outcomes, and prevent progression to advanced remodeling.

## Figures and Tables

**Table 1:** Clinical characteristics of patients treated with surgical and transcatheter aortic valve replacement.

Clinical Characteristics	SAVR (N = 350)	TAVR (N = 524)	*p* Value
Age—years	69.5 (9.5)	81.9 (6.1)	**<0.001**
Female sex	132 (37.7)	272 (52.4)	**<0.001**
EuroSCORE II	2.7 (3.3)	6.2 (7.0)	**<0.001**
Arterial hypertension	254 (72.8)	423 (80.9)	**0.005**
Diabetes	132 (37.7)	186 (35.8)	0.573
Smoking	48 (13.8)	127 (24.5)	**<0.001**
Chronic kidney disease	68 (19.4)	134 (25.9)	**0.028**
Obesity	26 (7.4)	167 (32.2)	**<0.001**
Atrial fibrillation	51 (14.6)	146 (28.6)	**<0.001**
Chronic obstructive pulmonary disease	41 (11.7)	72 (13.9)	0.353

Data are presented as mean (standard deviation) or frequency (percentage). Values in bold are significant. SAVR: surgical aortic valve replacement, TAVR: transcatheter aortic valve replacement.

**Table 2:** Clinical characteristics of intermediate and low surgical risk patients (EuroSCORE II < 8) treated with surgical and transcatheter aortic valve replacement.

Clinical Characteristics	SAVR (N = 330)	TAVR (N = 417)	*p* Value
Age—years	69.3 (9.5)	81.9 (5.8)	**<0.001**
Female sex	127 (38.5)	225 (54.5)	**<0.001**
EuroSCORE II	2.1 (1.4)	3.6 (1.8)	**<0.001**
Arterial hypertension	238 (72.3)	332 (80.4)	**0.010**
Diabetes	123 (37.3)	145 (35.1)	0.542
Smoking	44 (13.4)	99 (24.0)	**<0.001**
Chronic kidney disease	60 (18.2)	88 (21.4)	0.282
Obesity	25 (7.6)	134 (32.4)	**<0.001**
Atrial fibrillation	45 (13.6)	117 (28.8)	**<0.001**
Chronic obstructive pulmonary disease	34 (10.3)	50 (12.1)	0.440

Data are presented as mean (standard deviation) or frequency (percentage). Values in bold are significant. SAVR: surgical aortic valve replacement, TAVR: transcatheter aortic valve replacement.

**Table 3:** Echocardiographic findings of patients treated with surgical and transcatheter aortic valve replacement.

Echocardiographic Findings	SAVR (N = 350)	TAVR (N = 524)	*p* Value
Peak AV velocity—m/s	4.4 (0.7)	4.3 (0.6)	**0.013**
Peak AV gradient—mmHg	80.6 (23.1)	75.2 (21.4)	**0.000**
Mean AV gradient—mmHg	47.3 (14.8)	45.3 (13.9)	**0.044**
LVEDVI—mL/m^2^	54.4 (21.2)	56.0 (19.3)	0.267
LVMI—g/m^2^	122.3 (37.2)	126.2 (31.0)	0.109
LVEF—%	60.1 (10.5)	57.3 (10.2)	**<0.001**
LV-GLS—%	−15.0 (5.5)	−14.6 (4.4)	0.340
LAVI—mL/m^2^	37.1 (14.2)	50.6 (34.5)	**<0.001**
E/e’ ratio	14.1 (7.2)	15.2 (6.1)	**0.040**
Moderate/severe MR	34 (9.8)	88 (17.7)	**0.001**
Moderate/severe TR	27 (7.8)	74 (15.2)	**0.001**
TAPSE—mm	21.6 (4.8)	20.4 (4.5)	**0.001**
PASP—mmHg	26.9 (13.6)	36.7 (14.7)	**<0.001**
RVAc—mm/mmHg	1.0 (0.4)	0.7 (0.3)	**<0.001**

Data are presented as mean (standard deviation) or frequency (percentage). Values in bold are significant. AV: aortic valve, LAVI: left atrial volume index, LVEDVI: left ventricular end-diastolic volume index, LVEF: left ventricular ejection fraction, LV-GLS: left ventricle global longitudinal strain, LVMI: left ventricle mass index, MR: mitral regurgitation, PASP: pulmonary artery systolic pressure, RVAc: right ventricular–arterial coupling, SAVR: surgical aortic valve replacement, TAPSE: tricuspid annular plane systolic excursion, TAVR: transcatheter aortic valve replacement, TR: tricuspid regurgitation.

**Table 4:** Echocardiographic findings of intermediate and low surgical risk patients (EuroSCORE II < 8) treated with surgical and transcatheter aortic valve replacement.

Echocardiographic Findings	SAVR (N = 330)	TAVR (N = 417)	*p* Value
Peak AV velocity—m/s	4.4 (0.7)	4.3 (0.6)	0.062
Peak AV gradient—mmHg	81.0 (23.1)	76.5 (21.1)	**0.006**
Mean AV gradient—mmHg	47.6 (14.8)	46.4 (13.8)	0.284
LVEDVI—mL/m^2^	53.5 (19.4)	54.0 (16.7)	0.726
LVMI—g/m^2^	120.9 (36.0)	123.4 (28.4)	0.304
LVEF—%	60.8 (9.4)	59.2 (8.4)	**0.014**
LV-GLS—%	−15.3 (5.3)	−15.3 (3.9)	0.944
LAVI—mL/m^2^	36.8 (14.2)	48.5 (22.1)	**<0.001**
E/e’ ratio	14.0 (7.1)	15.1 (6.0)	0.055
Moderate/severe MR	27 (8.3)	52 (13.0)	**0.041**
Moderate/severe TR	26 (7.9)	48 (12.2)	0.059
TAPSE—mm	21.8 (4.6)	20.7 (4.4)	**0.001**
PASP—mmHg	26.3 (13.0)	35.4 (14.1)	**<0.001**
RVAc—mm/mmHg	0.98 (0.4)	0.72 (0.3)	**<0.001**

Data are presented as mean (standard deviation) or frequency (percentage). Values in bold are significant. AV: aortic valve, LAVI: left atrial volume index, LVEDVI: left ventricular end-diastolic volume index, LVEF: left ventricular ejection fraction, LV-GLS: left ventricle global longitudinal strain, LVMI: left ventricle mass index, MR: mitral regurgitation, PASP: pulmonary artery systolic pressure, RVAc: right ventricular–arterial coupling, SAVR: surgical aortic valve replacement, TAPSE: tricuspid annular plane systolic excursion, TAVR: transcatheter aortic valve replacement, TR: tricuspid regurgitation.

**Table 5:** Multivariable Cox regression analysis for all-cause mortality one year after the intervention.

**Model 1—Gutiérrez system**	HR (95% CI)	Adjusted *p* value
Age—years	1.04 (1.02–1.08)	**0.002**
Chronic kidney disease	2.62 (1.67–4.13)	**<0.001**
Right-sided cardiac damage by Gutiérrez classification	1.50 (1.22–1.86)	**<0.001**
**Model 2** **—** **Généreux system**	HR (95% CI)	Adjusted *p* value
Age—years	1.05 (1.02–1.08)	**0.001**
EuroSCORE II	1.04 (1.01–1.06)	**0.02**
Chronic kidney disease	2.91 (1.89–4.47)	**<0.001**
Right-sided cardiac damage by Généreux classification	1.05 (0.91–1.22)	0.487

Multivariable analyses were built adjusting for variables found to be significant on univariable Cox regression (as shown in Supplementary Table S3): age, atrial fibrillation, chronic renal disease, chronic obstructive pulmonary disease, EuroSCORE II, LVEF, and right-sided cardiac damage. Right-sided damage, as defined by the two different staging systems, was alternatively included in multivariable analyses. Values in bold are statistically significant. VIF for model 1: 1.03, VIF for model 2: 1.08.

## Data Availability

Dataset available on request from the authors due to restrictions (patients’ privacy).

## Supplementary Materials

The following supporting information can be downloaded at: https://www.mdpi.com/article/10.3390/jcm15051961/s1, Table S1: Clinical characteristics of low surgical risk patients (EuroSCORE II < 4) treated with surgical and transcatheter aortic valve replacement; Table S2: Echocardiographic findings of low surgical risk patients (EuroSCORE II < 4) treated with surgical and transcatheter aortic valve replacement; Table S3: Univariable Cox regression analysis for all-cause mortality one year after the intervention.
