# Supplementary material for: Aortic Stenosis-Associated Cardiac Damage: A Comparison Between Patients Treated with Surgery and Transcatheter Aortic Valve Replacement
Source: J Clin Med. 2026 Mar 4;15(5):1961. doi: 10.3390/jcm15051961 (PMC12985492; doi:10.3390/jcm15051961)
Supplement: Supplementary file 1 [file jcm-15-01961-s001.zip › jcm-4149241-supplementary.pdf]

SUPPLEMENTARY MATERIAL

Supplementary Table S1. Clinical characteristics of low surgical risk patients (EuroSCORE II <4) treated with surgical and transcatheter aortic valve replacement.

Clinical characteristics	SAVR (N = 295)	TAVR (N = 260)	P value
Age – years	68.8 (9.4)	81.6 (5.8)	**<0.001**
Female sex	115 (39.0)	129 (49.6)	**0.012**
EuroSCORE II	1.7 (0.9)	2.4 (0.9))	**<0.001**
Arterial hypertension	210 (71.4)	206 (79.2)	**0.034**
Diabetes	104 (35.3)	80 (30.8)	0.263
Smoking	37 (12.6)	62 (23.9)	**0.001**
Chronic kidney disease	49 (16.6)	44 (17.0))	0.905
Obesity	23 (7.8)	85 (32.7)	**<0.001**
Atrial fibrillation	35 (11.9)	68 (26.7)	**<0.001**
Chronic obstructive pulmonary disease	28 (9.5)	33 (12.7)	0.229

Data are presented as mean (standard deviation) or frequency (percentage). Values in bold are significant. SAVR: surgical aortic valve replacement, TAVR: transcatheter aortic valve replacement.

Supplementary Table S2. Echocardiographic findings of low surgical risk patients (EuroSCORE II <4) treated with surgical and transcatheter aortic valve replacement.

Echocardiographic findings	SAVR (N = 295)	TAVR (N = 260)	P value
Peak AV velocity - m/s	4.5 (0.6)	4.4 (0.6)	0.274
Peak AV gradient– mmHg	81.9 (22.6)	78.3 (21.6)	0.060
Mean AV gradient– mmHg	48.0 (14.6)	48.1 (4.3)	0.957
LVEDVI - ml/m^2	53.0 (18.9)	53.9 (16.0)	0.572
LVMI - g/m^2	119.7 (35.2)	122.5 (27.0)	0.325
LVEF - %	61.6 (8.7)	59.8 (7.9)	**0.013**
LV-GLS - %	-15.7 (5.0)	-15.6 (3.8)	0.801
LAVI - ml/m^2	36.0 (13.1)	44.7 (17.6)	**<0.001**
E/e´ ratio	13.5 (5.9)	14.9 (6.1)	**0.025**
Moderate/severe MR	22 (7.5)	23 (9.2)	0.484
Moderate/severe TR	24 (8.2)	24 (9.8)	0.516
TAPSE – mm	22.0 (4.5)	21.3 (4.4)	0.093
PASP – mmHg	25.9 (12.9)	33.2 (12.2)	**<0.001**
RVAc - mm/mmHg	1.0 (0.4)	0.76 (0.3)	**<0.001**

Data are presented as mean (standard deviation) or frequency (percentage). Values in bold are significant. AV: aortic valve, LAVI: left atrial volume index, LVEDVI: left ventricular end-diastolic volume index, LVEF: left ventricular ejection fraction, LV-GLS: left ventricle global longitudinal strain, LVMI: left ventricle mass index, MR: mitral regurgitation, PASP: pulmonary artery systolic pressure, RVAc: right ventricular-arterial coupling, SAVR: surgical aortic valve replacement TAPSE: tricuspid annular plane systolic excursion, TAVR: transcatheter aortic valve replacement, TR: tricuspid regurgitation.

Supplementary Table S3. Univariable Cox regression analysis for all-cause mortality one year after the intervention.

	HR (95% CI)	p value
Age – years	1.06 (1.04-1.09)	**<0.001**
Female sex	1.31 (0.88-1.94)	0.179
EuroSCORE II	1.06 (1.04-1.08)	**<0.001**
Arterial hypertension	2.24 (1.22-4.10)	**0.009**
Diabetes	1.24 (0.83-1.85)	0.293
Smoking	1.01 (0.61-1.67)	0.964
Chronic kidney disease	3.80 (2.56-5.64)	**<0.001**
Obesity	1.85 (1.21-2.82)	**0.004**
Atrial fibrillation	1.95 (1.29-2.94)	**0.002**
Chronic pulmonary disease	1.71 (1.04-2.82)	**0.034**
Left ventricular ejection fraction	0.97 (0.96-0.99)	**0.003**
Right-sided cardiac damage by Gutiérrez classification	2.50 (1.50-4.16)	**<0.001**
Right-sided cardiac damage by Généreux classification	1.56 (1.03-2.36)	**0.035**

Statistically significant results are shown in bold. Hazard ratios (HR) and 95% confidence intervals (CI) are presented.
